# Simulation of SAW Humidity Sensors Based on $\left(11\bar{2}0\right)$ZnO/R-Sapphire Structures

**DOI:** 10.3390/s16111112

**Published:** 2016-11-02

**Authors:** Xiao-Dong Lan, Shu-Yi Zhang, Li Fan, Yan Wang

**Affiliations:** 1 Laboratory of Modern Acoustics, Institute of Acoustics, Collaborative Innovation Center of Advanced Microstructures, Nanjing University, Nanjing 210093, China; lanxiaodong@nju.edu.cn (X.-D.L.); FanLi@nju.edu.cn (L.F.); ywang@njupt.edu.cn (Y.W.); 2 College of Electronic Engineering, Nanjing University of Posts and Telecommunications, Nanjing 210046, China

**Keywords:** humidity sensor, surface acoustic wave, Rayleigh wave, Love wave, FEM simulation

## Abstract

The characteristics of two types of surface acoustic waves SAWs (Rayleigh waves and Love waves) propagating in bilayered structures of $\left(11\bar{2}0\right)$ZnO/R-sapphire are simulated by a finite element method (FEM) model, in which both SAWs have crossed propagation directions. Furthermore, based on the bilayered structures, the frequency responses of Rayleigh wave and Love wave humidity sensors are also simulated. Meanwhile, the frequency shifts, insertion loss changes and then the sensitivities of both humidity sensors induced by the adsorbed water layer perturbations, including the mechanical and electrical factors, are calculated numerically. Generally, the characteristics and performances of both sensors are strongly dependent on the thickness of the ZnO films. By appropriate selecting the ratio of the film thickness to SAW wavelength for each kind of the sensors, the performances of both sensors can be optimized.

## 1. Introduction

Surface acoustic waves SAW-based sensors have gained increasing attention in a wide range of applications because of their small size, high sensitivity and good reaction speed for variations of environmental conditions. Various SAW sensors had been developed and applied to physical, chemical, industrial and biomedical fields [1,2,3,4,5]. Meanwhile, to fabricate SAW sensors with high sensitivity and low temperature coefficients, multilayered structures were generally selected, in which ZnO films deposited on substrates were preferred layered materials [3,4,6,7]. In previous studies, several kinds of bilayered structures based on ZnO thin films deposited on sapphire substrates had been investigated and reported in order to achieve high SAW velocities and low temperature coefficients [8,9]. Considering SAWs with SH-modes are more suitable for sensing in liquid environments, a kind of Love mode sensors based on the bilayered structures of$\;\left(11\bar{2}0\right)$ZnO thinilmssputteredon R-sapphire substrates had been prepared and investigated experimentally [10,11]. 

On the other hand, following the developments of the techniques and applications of SAW sensors, the related theoretical investigations had also attracted a great attention. To calculate the propagation characteristics of SAWs, and also the frequency responses and sensitivities of SAW sensors, the transfer matrix method for numerical calculations [12,13,14] and finite element method (FEM) for numerical simulations [15,16] had been always used by several authors. 

For example, Mitsuyu et al. [8] and then Wang et al. [9] calculated the characteristics of the SAWs propagating in the bilayered structure of $\left(11\bar{2}0\right)$ZnO/R-sapphire by the transfer matrix method. The calculated results indicated that layered structures can be used to excite SAWs with two different modes (Rayleigh mode and Love mode) propagating in two different directions. The effective electromechanical coupling coefficients exhibit a maximum in the (0001) direction for Rayleigh-type waves and the $\left(1\bar{1}00\right)$ direction for Love type waves. Therefore, the Rayleigh wave and Love wave sensors can be fabricated simultaneously on the structures of $\left(11\bar{2}0\right)$ZnO/R-sapphire [14,15], but with different propagation directions. 

In this paper, the propagation characteristics of SAWs (Rayleigh wave and Love wave) based on the structures of $\left(11\bar{2}0\right)$ZnO/R-sapphire are simulated firstly by a 3-D finite element method (FEM) multi-physics simulation model of COMSOL 5.0. Then, the frequency responses and sensitivities of humidity sensors with Rayleigh modes and Love modes based on the structures of $\left(11\bar{2}0\right)$ZnO/R-sapphire are also simulated numerically. The simulation results agree well with previous published experimental and theoretical data [8,9,10]. Besides, the mechanical and electrical contributions of the adsorbed water layer to the sensitivities are simulated and discussed. According to the simulated results, it is illustrated that the FEM simulation will provide an effective method to design SAW humidity sensors and evaluate the performance and suitability of the sensors.

## 2. Materials and Structures

**Figure 1:**
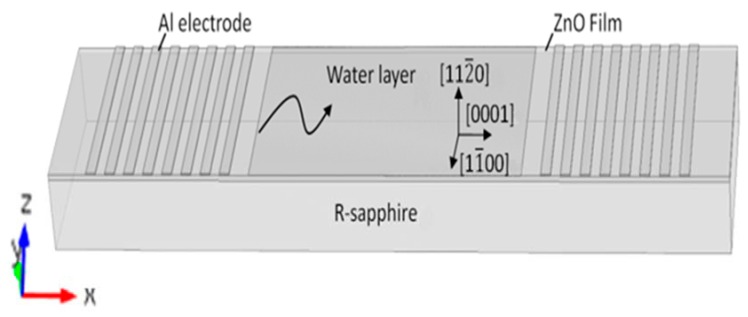
Schematic diagram of the Rayleigh wave sensor.

A model of SAW sensors with multi-layered structure is shown in Figure 1, in which a R-sapphire plate is used as a substrate and a $\left(11\bar{2}0\right)$ ZnO thin film is deposited on the substrate. Based on previous theoretical calculations and experiments [8,9], for the bilayered structures $\left(11\bar{2}0\right)$ZnO/R-sapphire, there are two modes can be excited effectively in different propagation directions, i.e., Rayleigh wave propagates in the direction (0001) and Love wave propagates in the direction $\left(1\bar{1}00\right)$. Therefore, two kinds of SAW sensors can be fabricated by suitably selecting the positions of interdigital transducers (ITD) and propagation directions of SAWs. 

At first, the vibration modes, propagation velocities and related electromechanical coupling coefficients of the SAW propagating in the bi-layered $\left(11\bar{2}0\right)$ZnO/R-sapphire structures are simulated with the 3-D FEM model in COMSOL 5.0.

In the numerical analysis, the model of the sensor shown in Figure 1 is meshed with triangular elements. The mesh density for the device is kept as 16 nodes per wavelength (i.e., 1 node for 1 μm), but if the surface of the device is covered by a water layer induced by adsorption of water vapor, the mesh density in water layer is decreased to five nodes per wavelength. 

The boundary conditions of the device are as follows: the top surface is free of mechanical boundaries, and the others are low-reflecting mechanical boundaries. For the water layer adsorbed on the surface of the humidity sensor, soft boundary is used. Besides, all of the boundaries are with zero charge for electrical boundary conditions, and all of the interfaces are with continuous boundary conditions. The material parameters are listed in Table 1.

## 3. Characteristics of SAW Propagating in $\left(11\bar{2}0\right)$ZnO/R-Sapphire Structures

**Figure 2:**
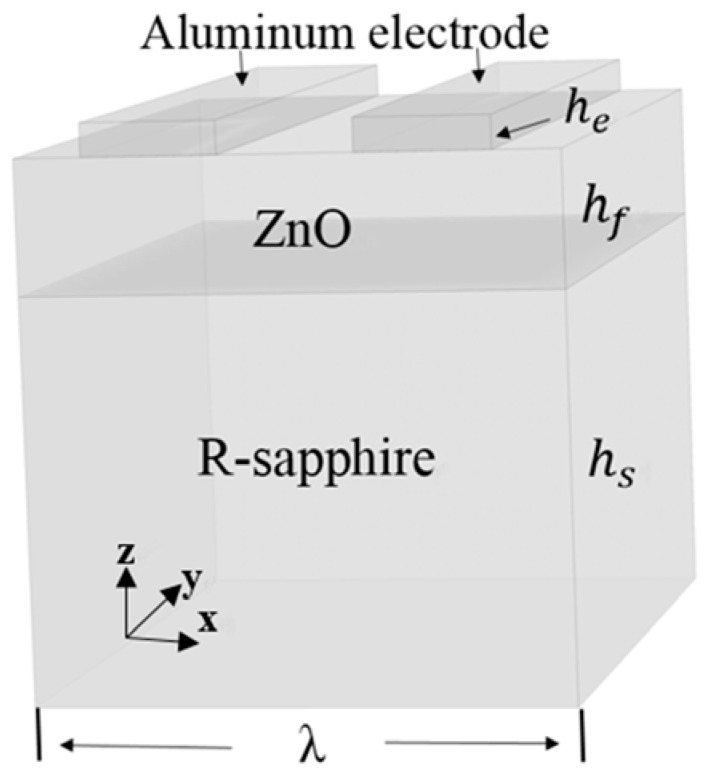
Schematic diagram of bi-layered structure $\left(11\bar{2}0\right)$ZnO/R-sapphire and electrodes.

To simulate the vibration modes excited by an IDT with the same electrode shape, for simplicity, which is excited by one period of the IDT, but the left and right sides of the IDT have periodical boundary conditions, is considered [17], so the physical model consisting of a R-sapphire substrate, a $\left(11\bar{2}0\right)$ZnO film and a pair of Al film electrodes of IDT is schematically shown in Figure 2. The thicknesses of the Al electrodes, ZnO film and R-sapphire substrate are denoted by *h_e_*, *h_f_* and *h_s_* respectively, and the wavelength of the SAW is denoted by λ, and the structure parameters are: *h_e_* = 0.2 μm, *h_f_* = 1.76 μm, *h_s_* = 100 μm and λ = 16 μm. 

Based on the material constants and the structure parameters, the characteristics of the SAW propagating in the bilayered $\left(11\bar{2}0\right)$ZnO/R-sapphire structures are simulated with the 3-D FEM model by COMSOL 5.0. 

**Figure 3:**
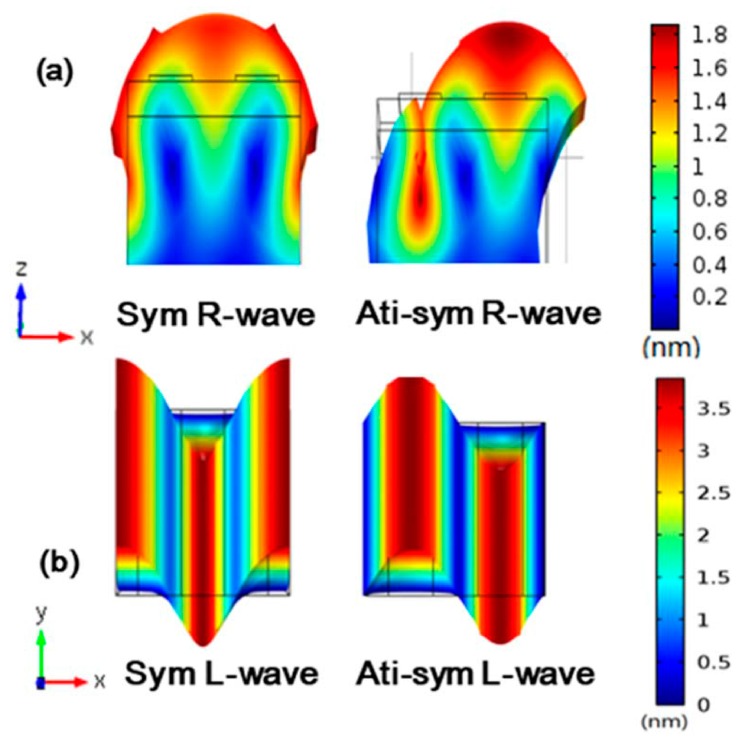
Displacement distributions in symmetric and anti-symmetric modes: (**a**) Raleigh wave; (**b**) Love wave.

Then, the eigenmodes of the Rayleigh wave and Love wave propagating in the (0001) direction and $\left(1\bar{1}00\right)$ direction of the substrate are simulated, respectively. The displacements of the Raleigh mode and Love mode resulting from the physical model (Figure 2) are shown in Figure 3. 

For each wave, there are two eigenfrequencies, $f_{M-}$ for symmetric and $f_{M+}$ for anti-symmetric, then the resonance frequency $\;f_{0}\;$can be calculated by [17]:

(1) $$\;f_{0}=\left(f_{M-}+f_{M+}\right)/2$$

Furthermore, from the FEM simulations, the velocities of the SAWs corresponding free- and shorted-electrical surface boundary conditions, *v_o_* and *v_s_*, are calculated with multiplying the wavelength by related resonance frequencies, respectively. Then, based on the corresponding *v_o_* and *v_s_*, the electromechanical coupling coefficients (*k*^2^) of the structures can be calculated by:

(2) $$\;k^{2}=\frac{2\left(v_{o}-v_{s}\right)}{v_{o}}$$

**Figure 4:**
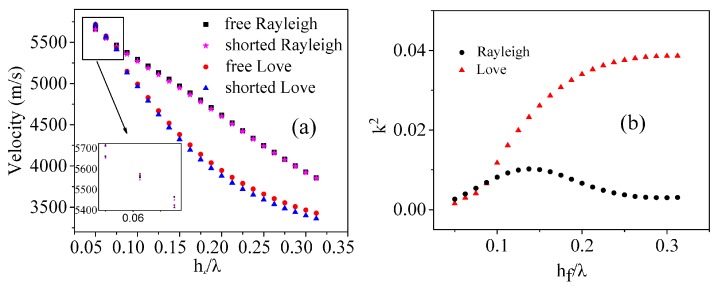
Velocity and electromechanical coupling coefficient of Rayleigh and Love waves vs thickness of ZnO film: (**a**) velocity; (**b**) electromechanical coupling coefficient.

The velocities and electromechanical coupling coefficients corresponding to Rayleigh and Love modes for different thicknesses *h_f_* of ZnO film, but with the same thicknesses of *h_e_* = 0.5 μm, *h_s_* = 100 μm and wavelength λ = 16 μm, are shown in Figure 4a,b, respectively. 

The calculated results agree well with those obtained previously by the transfer matrix theories and experiments for both different waves [8,9].

## 4. Performances of the SAW Humidity Sensors

For studying the performances of both Rayleigh wave and Love wave sensors based on the $\left(11\bar{2}0\right)$ZnO/R-sapphire structures, the sensors for humidity are simulated as follows:

(1) Frequency responses of sensors

To evaluate the frequency response of the humidity sensor, an electrical signal *V*_input_ with Gaussian pulse is applied to excite an IDT. Then, the output signal *V*_output_ can be simulated. In the FEM simulations, the ZnO film thickness is selected as *h_f_* = 1.76 μm for λ = 16 μm (i.e., *h_f_*/λ = 0.11), which is generally the thickness for obtaining optimized electromechanical coupling coefficient of Rayleigh waves. The sizes in *X*, *Y* and *Z* directions of the substrates are 280 μm, 64 μm and 32 μm, respectively. The distance between both IDTs is 128 μm, and each of the IDTs is composed of four pairs of Al electrodes with 64 μm aperture, the same electrode width of 4 μm and metalized ratio of 1. Thus the insertion loss (IL) can be calculated by:

(3) $$\text{IL}=20\times \log_{10}\left|(V_{\text{output}}-V_{\text{input}})/V_{\text{input}}\right|$$

**Figure 5:**
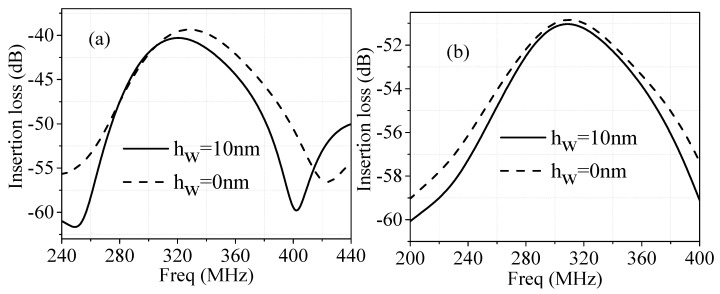
Frequency responses of humidity sensors: (**a**) Rayleigh wave; (**b**) Love wave.

The simulated frequency responses (IL) for both Rayleigh wave and Love wave sensors are shown (dashed lines) in Figure 5a,b, respectively.

As water vapor is adsorbed on the sensing surface of the sensor, the *V*_output_ of receiving IDT varies with the water layer perturbation, which can be quantified as the characteristics of the frequency shift (Δf) and insertion loss change (ΔIL). In the simulation, the meshed element density is decreased to 5 nodes per wavelength in water layer. The water layer is with the area as 112 × 64 μm^2^, which is apart λ/2 from each IDT, and with the thickness *h_w_*. 

Generally, for humidity sensors, the Δf and/or ΔIL are controlled by two factors of the mass loading and conductivity variation due to the adsorbed water layer, which are related to the parameters: density $\rho_{w}$, dielectric constant $\varepsilon_{w}$ and thickness of the water layer *h_w_*. In the simulation, $\rho_{w}$ = 1000 kg/m^3^, but $\varepsilon_{w}$ is dispersive in the frequency range 240–440 MHz for SAW propagation due to the relaxation effect of water [18]. Considering the SAW devices are with very narrow bandwidths in our case, the central frequencies are selected in the range of 300–320 MHz and bandwidth about 80 MHz, then $\varepsilon_{w}$ can be selected as about 17 (much less than that of normal cases, 80) [18]. Thus, the simulated frequency responses of the Rayleigh wave and Love wave humidity sensors over the frequency range of 200–440 MHz are also shown in Figure 5a,b (solid lines).

**Figure 6:**
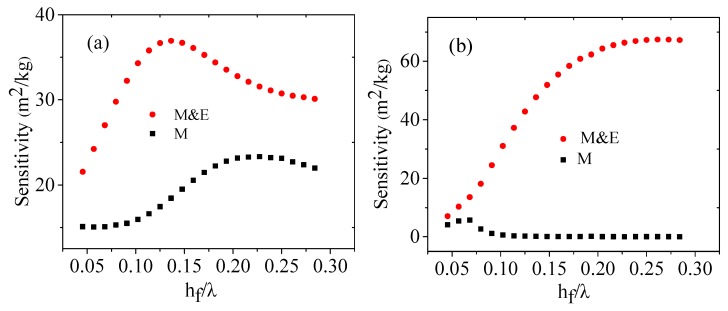
Sensitivities of humidity sensors induced by mass loading (M) and mass loading & conductivity variation (M & E) for: (**a**) Rayleigh wave; (**b**) Love wave.

From Figure 5a, it can be seen, when the thickness (*h_w_*) of the water layer is 10 nm, the central frequency of the sensor decreases from 328 MHz to 320 MHz and the insertion loss changes from −39.36 dB to −40.31 dB with the central frequency variations. For the Love wave sensor, as shown in Figure 6b, when the *h_w_* is also 10 nm, the central frequency shifts approximately from 310 MHz to 309 MHz, and the insertion loss changes from −50.76 dB to −51.04 dB.

(2) Sensitivities of sensors

For the sensing mechanism of SAW sensors, it is always just considered the responses of the mass loading (Δm) on the device surface. The mass sensitivity S*_m_* of SAW sensor is calculated by:

(4) $$S_{m}=\underset{\Delta \text{m}\to 0}{\lim}\frac{1}{\Delta m}\frac{\Delta f}{f_{0}}$$

where $\Delta m=\rho_{w}\Delta h_{w}$ is induced by the small variation of the thickness for the water layer.

Practically, the mechanical loading and electrical property (or conductivity) variations of the water layer induce simultaneously the frequency shifts and insertion loss variations of the sensors, so it is needed to consider both of them together for the sensitivity calculations of the sensors [19]. Thus, the sensitivity can be denoted as the sum of both effects, then the total sensitivity S can be expressed as:

(5) $$S=S_{m}+S_{c}=\underset{\Delta \text{m}\to 0}{\lim}\frac{1}{\Delta m}\frac{\Delta f}{f_{0}}+\underset{\Delta \text{c}\to 0}{\lim}\frac{1}{\Delta c}\frac{\Delta f}{f_{0}}$$

where S*_c_* is called “conductivity sensitivity” and Δc is the conductivity variation induced by the water layer, which is mainly related to the dielectric constant $\varepsilon_{w}$ of the water layer.

As $\Delta h_{w}=0.5\;\text{nm}$ and the ZnO film thickness *h_f_* is varied in the range of (0.8–5) μm (λ = 16 μm), the S and S*_m_* variations with *h_f_* (ZnO film thickness) for both Rayleigh wave and Love wave sensors are simulated as shown in Figure 6a,b, respectively. It shows that the mass sensitivity S*_m_* of the Rayleigh wave sensor is much higher than that of Love wave sensor, which illustrates that the normal displacements of Rayleigh wave is much more sensitive to water mass loading than the shear displacements of the Love wave. 

Figure 6 also shows that the total sensitivity “M & E” of the Rayleigh wave sensor is higher than that of Love wave in the range of *h_f_*/λ < 0.11, but as *h_f_*/λ > 0.11, the phenomena are opposite. Meanwhile, as *h_f_*/λ = 0.11, both sensitivities of Rayleigh and Love wave sensors are almost the same. The maxima of sensitivities for Rayleigh wave sensor and Love wave sensor appear at about *h_f_*/λ = 0.13 and *h_f_*/λ = 0.25, respectively. The results demonstrate that the contributions of conductivity variation induced by water molecules to the sensitivity are distinguishable, especially for the Love wave sensors. 

On the other hand, the variation trends of the sensitivities with the thickness of ZnO film for both Rayleigh wave sensor and Love wave sensor are similar to those of the electromechanical couple coefficients of the corresponding waves shown in Figure 4, which indicates that the electromechanical couple coefficients of the films may have important effects on the contributions of the conductivities.

## 5. Analyses for Perturbations of Water Layers on Humidity Sensors

**Figure 7:**
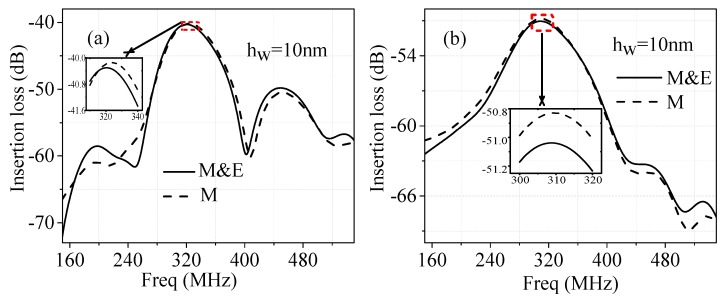
Frequency spectra of insertion loss induced by mechanical (M) and both mechanical and electrical (M & E) effects of the water layer: (**a**) Rayleigh wave sensor; (**b**) Love wave sensor.

To study the sensing characteristics of the SAW sensors based on the tri-layered structures of water layer/$\left(11\bar{2}0\right)$ZnO/R-sapphire in more details, the factors inducing the frequency response changes of the SAW sensors are analyzed. The frequency response spectra can also be separated into two parts: the mechanical (M) and electrical (E) affects. When the *h_w_* is 10 nm, considering the effects with and without the electrical properties, the frequency spectra of the Rayleigh wave and Love wave sensors are simulated as shown in Figure 7a,b, respectively. It is shown that the electrical affect of water layer to the insertion loss at around the center frequency for Rayleigh wave sensor is about 0.16 dB, while for Love wave sensor, is about 0.20 dB, which illustrate that the electrical affect for the Love wave sensor is bigger than that for the Rayleigh wave sensor.

**Figure 8:**
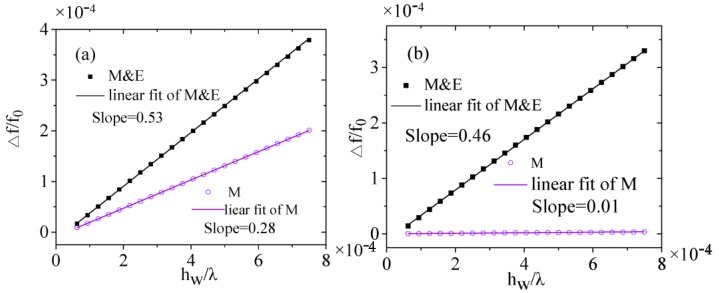
Mechanical and electrical contributions to frequency shift of humidity sensor: (**a**) Rayleigh wave; (**b**) Love wave.

According to previous experimental and theoretical calculations, as the environmental humidity changes approximately from 0.01% to 90%, the corresponding water layer height on the surface of the humidity sensor changes from 1 nm to 12 nm [10,18]. The frequency shifts Δ*f*/*f*_0_ of the SAW humidity sensors (*h*_f_/λ = 0.11) against different water layer heights *h_w_*/λ are also simulated, which are obtained by the resonant frequency evaluations using Equation (1) with and without water layers as shown in Figure 8a,b for Rayleigh waves and Love waves, respectively. Figure 8a,b show that the (Δ*f*/*f*_0_) changing against $h_{w}/\lambda$ can be fitted linearly, therefore as the water layer height is very small (*h_w_* ≪λ), the relationships between the (Δ*f*/*f*_0_) and $h_{w}/\lambda$, can be expressed as:

(6) $$-\frac{\Delta f}{f}=\left(A_{i}+B_{i}\right)\frac{h_{w}}{\lambda }$$

where $A_{i}$ and $B_{i}$ (*i* can be R or L to denote Rayleigh wave or Love wave ) represent mechanical and electrical factors induced by absorbed water layer, respectively. The simulated relationships are in agreement with the results of [18]. 

In addition, due to the adsorbed water layer height is always not proportional to the environmental relative humidity [10,18], so Δ*f*/*f*_0_ is also not changed linearly with the relative humidity variation.

From Figure 8, it can be calculated that, for the Rayleigh wave sensor, A_R_ = 0.28, B_R_ = 0.25, and for Love wave sensor, A_L_ = 0.58 × 10^−2^, B_L_ = 0.45. These results also demonstrate that, for Love wave, the mechanical effect is very small, since the vibration mode does not contain perpendicular displacement and almost no energy radiates into the liquid-like layers above the devices. Therefore, the frequency shift of the Love wave humidity sensor is mainly perturbed by the electrical property of water layer. On the other hands, the Rayleigh wave sensors are also sensing for water layer with almost the same sensitivity as the Love wave ones. But due to the perpendicular displacements loss of Rayleigh waves in water layer, Rayleigh wave sensors are not stable, and then not suitable to be used for humidity sensing as described by the previous experiments [11].

In addition, it must be indicated the simulated sensitivities of both sensors are very low, which are induced by the reduced parameters of the IDTs, such as with less numbered electrodes and small sizes of the aperture, and distance between both IDTs in order to decrease the simulation time as well.

## 6. Conclusions

In this work, the characterizations of Rayleigh wave and Love wave humidity sensors based on the $\left(11\bar{2}0\right)$ZnO/R-sapphire structures are simulated by the FEM model with COMSOL 5.0 software. At first, the electromechanical couple coefficients of the Rayleigh wave and Love wave in the structures $\left(11\bar{2}0\right)$ZnO/R-sapphire at different heights of ZnO films are calculated. Further, the variation trends of the sensitivities with the thickness of ZnO films for both the Rayleigh wave and Love wave sensors are also evaluated, which are similar to that of the corresponding electromechanical couple coefficients. Finally, the mechanical and electrical contributions of water layer perturbation to the frequency shifts and insertion loss changes of the SAW humidity sensors are calculated and discussed. The frequency shifts and insertion loss changes of both kinds of humidity sensors can be used to evaluate the water layer thickness corresponding to the humidity in the environment successfully. The results can provide more detailed understanding on the mechanisms of the humidity sensing of the SAW sensors and also provide valuable information to optimize the structures of the sensors. 

## Figures and Tables

**Table 1:** Material parameters ^a^.

Parameter	Symbol	ZnO	Al_3_O_2_	Al	Water
Density (kg/m^3^)	ρ	5680	3970	2700	1000
Poisson ratio	*v*			0.33	
Young’s modulus (10^9^ N/m^2^)	*E*			70	
Elastic constant (10^9^ N/m^2^)	*C*_11_	209.7	494		
	*C*_12_	121.1	158		
	*C*_13_	105.3	114		
	*C*_14_		−230		
	*C*_33_	211.2	496		
	*C*_44_	423.7	145		
Piezoelectric constants (C/m^2^)	*e*_15_	−0.481			
	*e*_31_	−0.567			
	*e*_33_	1.320			
Dielectric constant	$\varepsilon_{11}/\varepsilon_{0}$	8.55		9.34	80
	$\varepsilon_{33}/\varepsilon_{0}$	10.2		11.54	
Acoustic speed					1450

^a^ From COMSOL 5.0 MATERIAL LIBRARY.
